# Volatile Anesthetic-Based Combination Strategies Against Ischemia–Reperfusion Injury: Experimental Evidence, Molecular Mechanisms, and Translational Limitations

**DOI:** 10.3390/ijms27209335

**Published:** 2026-10-21

**Authors:** Necmiye Şengel, Mustafa Arslan

**Affiliations:** 1 Department of Oral and Maxillofacial Surgery, Faculty of Dentistry, Gazi University, Ankara 06490, Türkiye; necmiyesengel@gazi.edu.tr; 2 Department of Anesthesiology and Reanimation, Faculty of Medicine, Gazi University, Ankara 06560, Türkiye; 3 Life Sciences Application and Research Center, Gazi University, Ankara 06830, Türkiye; 4 Laboratory Animal Breeding and Experimental Research Center (GÜDAM), Gazi University, Ankara 06510, Türkiye

**Keywords:** ischemia–reperfusion injury, volatile anesthetics, sevoflurane, desflurane, fullerenol C60, ischemic conditioning, ozone, boric acid, combination therapy

## Abstract

Ischemia–reperfusion (I/R) injury is a complex pathological process involving adenosine triphosphate (ATP) depletion, calcium overload, excessive reactive oxygen species generation, mitochondrial dysfunction, inflammatory activation, and regulated cell death. Volatile anesthetics, including sevoflurane, desflurane, and isoflurane, may modulate several of these processes through antioxidant and prosurvival signaling, suppression of inflammatory pathways, and regulation of apoptosis. Because I/R injury involves interconnected mechanisms, combining interventions that act on different targets may offer additional protection. Experimental studies have investigated volatile anesthetics together with fullerenol C60, ischemic conditioning, and selected pharmacological interventions. Other combination strategies involving ozone conditioning and boric acid have also been evaluated in experimental models, although these have not established the effects of combining either intervention with a volatile anesthetic. This narrative review examines the molecular mechanisms and organ-specific evidence underlying these approaches, with particular attention to study design, treatment timing, and the interpretation of combined effects. While some combinations have shown greater protection than individual interventions, the findings remain heterogeneous, and formal analyses distinguishing additivity from synergy are generally lacking. Clinical evidence is limited, and further studies are needed to establish whether these experimental findings can be translated into meaningful patient outcomes.

## 1. Introduction

Ischemia–reperfusion (I/R) injury occurs when restoration of blood flow to previously ischemic tissue initiates a second phase of cellular damage. Although reperfusion is essential for tissue survival, the abrupt return of oxygen and substrates promotes reactive oxygen species (ROS) generation, calcium overload, mitochondrial permeability transition, endothelial dysfunction, and recruitment of inflammatory cells. These events reinforce one another and may lead to apoptosis, necrosis, and other forms of regulated cell death [1]. The resulting injury is relevant not only to myocardial infarction and stroke but also to organ transplantation, vascular surgery, tourniquet application, and periods of intraoperative hypoperfusion.

A wide range of pharmacological and conditioning strategies has been investigated, yet their clinical effects remain inconsistent. One reason may be that many interventions act mainly on a single component of a networked injury response. Limiting oxidative stress, for example, may not be sufficient when mitochondrial failure, inflammation, and microvascular impairment continue in parallel. Combination treatment is therefore attractive because two interventions may act at different stages or on partly complementary pathways. It may also allow lower doses of each component. These potential advantages, however, must be balanced against overlapping mechanisms, ceiling effects, pharmacological interference, and additional safety concerns.

Volatile anesthetics are particularly relevant in this setting because they are already used during procedures in which I/R may occur. Sevoflurane, desflurane, and isoflurane have been associated with mitochondrial preservation, activation of prosurvival signaling, modulation of nuclear factor erythroid 2-related factor 2/heme oxygenase-1 (Nrf2/HO-1) and toll-like receptor 4 (TLR4)-related inflammatory pathways, and reduced apoptosis in experimental models [2,3,4,5,6]. Their combination with redox-active compounds or ischemic conditioning may therefore broaden protection, but the available studies do not show a uniform advantage over either intervention alone [3,7,8,9,10,11,12,13].

This narrative review focuses primarily on experimental studies investigating volatile anesthetics in combination with fullerenol C60, ischemic conditioning, or selected pharmacological interventions for protection against I/R injury. These approaches were selected because they have been evaluated directly in combination-treatment models, allowing comparison with individual interventions. Ozone conditioning and boric acid-based combinations are discussed separately as related experimental strategies with potentially relevant protective mechanisms, rather than as established adjuncts to volatile anesthetics. Among boron-containing compounds, only boric acid [B(OH)_3_] has been evaluated in these combination models; organoboron drugs such as bortezomib and other boron compounds are outside the scope of this review. Particular attention is given to experimental design, intervention timing, organ-specific responses, and whether reported findings support additional protection or a formally demonstrated interaction. To our knowledge, this is the first review to compare directly tested volatile anesthetic-based combinations against their single-intervention arms and to appraise claims of additivity and synergy against formal reference models. By separating directly tested combinations from potential future approaches, it also identifies the questions that remain open before clinical translation, including dose equivalence, treatment timing, and the absence of interaction analyses.

## 2. Literature Search Strategy

This narrative review was based on critical evaluation of publications identified before submission in September 2026, supplemented by searches performed on 9 October 2026 during revision. The initial literature identification included PubMed and Google Scholar searches followed by examination of original publications. During revision, three PubMed queries addressed volatile anesthetics, fullerenol, ozone, boric acid, and related combination strategies; a ten-year publication-date filter was active in the recorded search results (98, 10, and 80 hits, respectively). Complementary Google Scholar searches yielded approximately 49, 5240, and 127 results for three topic-focused queries, without date restrictions. Publications older than ten years were identified through the undated Google Scholar searches and the initial pre-submission literature evaluation. These figures are overlapping query-level results, not deduplicated screening counts; Google Scholar numbers are approximate. The review did not use a prospectively registered systematic-review protocol.

For the analysis of direct combination evidence, priority was given to original studies evaluating combined treatment alongside an I/R control and individual interventions. Studies assessing single treatments, or comparing separate interventions without a combined-treatment group, were considered as supporting mechanistic or contextual evidence rather than direct evidence of combination effects. The evidence was examined with regard to experimental models, doses, schedules, comparator groups, molecular findings, and statistical analyses. Additional benefit versus one intervention was distinguished from formally demonstrated additivity or synergy. Because this was a narrative rather than systematic review, evidence was synthesized descriptively; no systematic study-selection flow is claimed.

## 3. Why Combination Therapy May Improve Organ Protection

Combination strategies may be relevant to I/R injury because ischemia and reperfusion involve distinct but interconnected mechanisms. Ischemia is associated with adenosine triphosphate (ATP) depletion, ionic imbalance, cellular swelling, and metabolic acidosis. Reperfusion may then amplify injury through oxidative stress, mitochondrial dysfunction, endothelial activation, and inflammation [1]. Interventions acting at different stages may therefore have complementary effects, although this possibility requires direct experimental evaluation [14].

A biologically plausible combination may target more than one component of the injury response. For example, one intervention may attenuate oxidative stress while another influences mitochondrial function, inflammatory signaling, or regulated cell death [14]. To establish whether the combination provides additional protection, experiments should compare the combined treatment with each intervention alone. Such comparisons do not, by themselves, distinguish additivity from synergy under an explicit reference model [15,16,17].

### 3.1. Complementary Molecular Targets

Volatile anesthetics may influence mitochondrial function, prosurvival signaling, redox-responsive pathways, and apoptosis-related processes [2,3,4,5,6,14]. Fullerenol C60 has antioxidant properties that could complement these effects, although the extent and mechanism of protection vary by formulation and experimental model [7,8,9,10,12,18]. Ozone conditioning has been investigated as a stimulus for adaptive antioxidant responses [19,20]. In separate I/R experiments, boric acid combined with propolis has been associated with changes in oxidative stress, inflammatory markers, and apoptosis-related outcomes [21]. The latter results should not be generalized to other boron-containing compounds or interpreted as evidence for a volatile anesthetic–boric acid combination.

Interpretation also depends on the selected endpoint. A combination may improve a biochemical marker without demonstrating additional histological or functional benefit. In the volatile anesthetic–fullerenol studies, the relative performance of individual and combined interventions varied across organs and outcomes [7,8,9,10,12]. Such findings may reflect differences in experimental models, dose, timing, pathway overlap, or statistical power; the available studies do not distinguish these possibilities reliably [14,15,16,17].

### 3.2. Timing of Combined Interventions

The timing of administration determines which stage of I/R injury an intervention can influence. Preconditioning precedes ischemia, perconditioning occurs during ischemia, and postconditioning begins at or shortly after reperfusion. Combining interventions administered at different stages could broaden the period of biological influence. However, this does not necessarily result in greater protection, as indicated by studies of ischemic and anesthetic conditioning [3,13].

Comparisons between studies are limited by differences in anesthetic concentration, exposure duration, adjunct administration, and treatment timing relative to reperfusion. These variables may also affect the predominant biological action of an intervention. Experimental reports should therefore specify the timing of each treatment in relation to ischemia and reperfusion and avoid attributing differences in outcome solely to the agents used [14].

### 3.3. Additive Effects and True Synergy

Additional benefit, additivity, and synergy are distinct concepts. Additional benefit indicates that a combination produces a more favorable outcome than either intervention alone. Additivity refers to the combined effect expected under a specified reference model, whereas synergy requires an effect exceeding that expectation. An arithmetic sum of individual protective effects is not a universally valid reference, particularly for bounded outcomes [15,16,17].

Different interaction models require different assumptions. Bliss independence estimates the expected combined effect under an assumption of independent action for appropriately defined fractional responses. Loewe additivity uses dose equivalence and individual dose–response relationships. An I/R control, each intervention alone, and a combination group can establish whether additional protection is observed; factorial analyses may test interaction on a specified effect scale. However, conventional between-group comparisons do not establish pharmacological synergy, and a nonsignificant interaction does not prove additivity [15,16,17].

Most direct combination studies discussed here used fixed treatment protocols and conventional comparisons between experimental groups. Their findings may support additional protection, similar efficacy, or no incremental benefit, but formal synergy claims generally cannot be established from the reported analyses. Accordingly, the interpretations in the following sections distinguish observed treatment differences from additivity or synergy under an explicitly specified reference model [15,16,17].

## 4. Molecular Targets of Combination Strategies

### 4.1. Oxidative Stress and Nrf2/HO-1 Signaling

**Figure 1:**
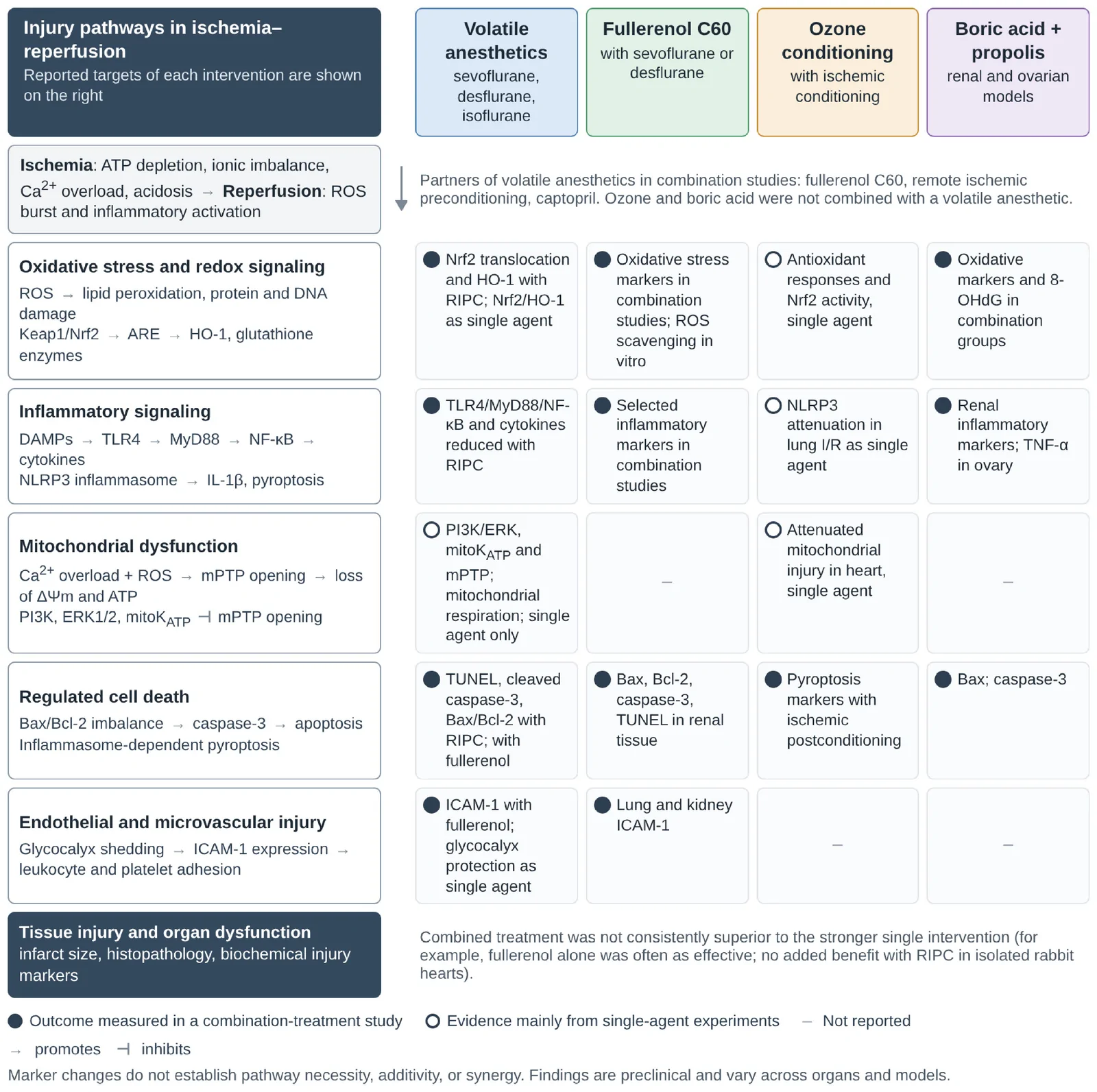
Proposed molecular targets of the reviewed interventions in ischemia–reperfusion injury. The left panel summarizes the principal injury pathways, and the right panel indicates the pathways on which each intervention has reported effects. Filled circles denote outcomes measured in combination-treatment studies; open circles denote evidence derived mainly from single-agent experiments. It does not imply that ozone or boric acid–propolis was combined with a volatile anesthetic, or that formal pharmacological synergy has been demonstrated. The findings are preclinical and vary across organs and models. Abbreviations: ATP, adenosine triphosphate; ROS, reactive oxygen species; Nrf2, nuclear factor erythroid 2-related factor 2; HO-1, heme oxygenase-1; TLR4, toll-like receptor 4; MyD88, myeloid differentiation primary response 88; NF-κB, nuclear factor kappa B; NLRP3, NLR family pyrin domain containing 3; mPTP, mitochondrial permeability transition pore; PI3K, phosphoinositide 3-kinase; ERK, extracellular signal-regulated kinase; ICAM-1, intercellular adhesion molecule-1; RIPC, remote ischemic preconditioning; ARE, antioxidant response element; DAMPs, damage-associated molecular patterns; Bax, Bcl-2-associated X protein; Bcl-2, B-cell lymphoma 2; TUNEL, terminal deoxynucleotidyl transferase dUTP nick end labeling; 8-OHdG, 8-hydroxy-2′-deoxyguanosine; TNF-α, tumor necrosis factor-α; IL-1β, interleukin-1β; mitoKATP, mitochondrial ATP-sensitive potassium channel; ΔΨm, mitochondrial membrane potential; I/R, ischemia–reperfusion; Keap1, Kelch-like ECH-associated protein 1. The supporting studies for each entry are cited in Section 4 and Section 5.

Oxidative stress contributes to I/R injury through ROS generation, lipid peroxidation, and damage to cellular proteins and DNA. These changes may accompany mitochondrial dysfunction and cell death, but individual biochemical markers should not be treated as direct measurements of organ function [1,14]. The principal injury pathways and the reported targets of each intervention are summarized in Figure 1.

Nrf2 regulates the cellular antioxidant response through interaction with Kelch-like ECH-associated protein 1 (Keap1) and antioxidant response elements (AREs). The associated genes include HO-1 and enzymes involved in glutathione metabolism. Increased Nrf2 or HO-1 expression is compatible with pathway engagement; establishing a necessary protective role generally requires additional functional experiments.

Ma et al. [2] reported that sevoflurane preconditioning attenuated hepatic I/R injury in rats and increased nuclear Nrf2 and HO-1 expression. In a complementary hepatocyte hypoxia–reoxygenation model, the Nrf2 inhibitor ML385 reduced sevoflurane-associated protection, providing functional support for pathway involvement. Zheng et al. [22] reported reduced renal injury and changes in Nrf2-Keap1-ARE-related proteins following desflurane exposure in a rat I/R model. Unlike Ma et al., this study did not establish pathway necessity through a targeted Nrf2 inhibition experiment. Both investigated volatile anesthetics without an additional organ-protective agent.

In isolated rat hearts, delayed remote ischemic preconditioning combined with sevoflurane postconditioning was associated with greater protection and higher HO-1 expression and nuclear Nrf2 translocation than the individual interventions [23]. However, the analysis did not establish a formal synergistic interaction or demonstrate that Nrf2 activation was required for the incremental protection.

Volatile anesthetic–fullerenol experiments reported improvements in selected oxidative and histological outcomes [7,8,9,10,12]. The combination studies did not directly investigate Nrf2/Keap1/HO-1 as the mediator of these effects, and combined treatment was not consistently superior to fullerenol alone. Related work on ozone conditioning and propolis with boric acid also reported favorable oxidative outcomes [19,21,24,25], but the mechanistic findings from single-treatment studies cannot be assumed to apply to untested combinations with volatile anesthetics.

### 4.2. Inflammatory Signaling

Inflammatory responses contribute to tissue injury during reperfusion. Damage-associated molecular patterns can stimulate TLR4 and downstream myeloid differentiation primary response 88 (MyD88) and nuclear factor kappa B (NF-κB) signaling, promoting the expression of inflammatory mediators [1]. Inflammasome activation and pyroptosis represent related, but distinct, processes in selected experimental models [19].

Zhang et al. [3] evaluated remote ischemic preconditioning combined with sevoflurane postconditioning in a rat myocardial I/R model. The combination reduced TLR4, MyD88, and NF-κB expression together with circulating inflammatory cytokines and was associated with greater protection than either intervention alone on several outcomes. However, pathway necessity was not directly examined through targeted inhibition in the combined-treatment setting, and the between-group analyses do not demonstrate pharmacological synergy.

Volatile anesthetic–fullerenol studies reported reductions in selected inflammatory markers, although the combined interventions did not consistently outperform fullerenol alone across organs and endpoints [7,8,9,10,12]. Combined ischemic and ozone postconditioning was associated with reductions in renal pyroptosis-related markers [19], whereas ozone preconditioning alone has been linked to reduced NLR family pyrin domain containing 3 (NLRP3)-associated inflammation in lung I/R [25]. These studies should not be treated as evidence that ozone has been combined with a volatile anesthetic.

Propolis with boric acid reduced renal inflammatory and oxidative injury markers in an experimental I/R model [21]. However, these outcomes do not isolate an inflammatory mechanism from concurrent antioxidant or histological effects. None of the cited direct-combination studies established inflammatory synergy under a prespecified additivity reference model.

### 4.3. Mitochondrial Dysfunction and Energy Failure

Mitochondrial dysfunction is a major component of I/R injury. During ischemia, reduced oxygen availability limits oxidative phosphorylation and depletes ATP. Reperfusion may promote calcium overload and excessive ROS generation, favoring opening of the mitochondrial permeability transition pore (mPTP). Sustained pore opening disrupts mitochondrial membrane potential and energy homeostasis and contributes to cell death. ATP synthase participates in proposed models of pore formation, although the precise molecular architecture remains under investigation [1,26].

Yao et al. [6] examined sevoflurane postconditioning in isolated rat hearts. Sevoflurane improved functional recovery and reduced infarct size; pharmacological inhibition of phosphoinositide 3-kinase (PI3K), extracellular signal-regulated kinase 1/2 (ERK1/2), or mitochondrial ATP-sensitive potassium channels, and pharmacological promotion of mitochondrial permeability transition, abolished these effects. Myocardial nicotinamide adenine dinucleotide (NAD+) content served as an indirect indicator of mPTP opening. Although these experiments support involvement of prosurvival and mitochondrial mechanisms, activation of PI3K and ERK1/2 was not directly confirmed by kinase phosphorylation measurements.

Xie et al. [5] reported cardioprotection with sevoflurane preconditioning in diabetic mice despite impaired AMP-activated protein kinase (AMPK) signaling, accompanied by increased ERK1/2 phosphorylation. Mitochondrial respiratory control ratio and maximal respiration also improved in normal, diabetic, and AMPK-impaired mice. These results provide direct functional mitochondrial evidence, but only for sevoflurane administered as a single intervention.

In contrast, the volatile anesthetic–fullerenol combination studies focused mainly on oxidative stress, inflammatory mediators, biochemical injury markers, and histopathology [7,8,9,10,12]. They did not establish improved mitochondrial respiratory function or reduced mPTP opening specifically attributable to the combinations. Likewise, no directly evaluated volatile anesthetic–ozone or volatile anesthetic–boric acid combination has demonstrated a mitochondrial interaction in the experimental evidence reviewed here. Mitochondrial preservation remains a plausible hypothesis that requires direct functional measurements and prespecified interaction analyses.

### 4.4. Apoptosis and Other Forms of Regulated Cell Death

Apoptosis contributes to I/R injury through pathways involving caspase activation and the balance between proapoptotic and antiapoptotic proteins, such as Bcl-2-associated X protein (Bax) and B-cell lymphoma 2 (Bcl-2). Ferroptosis, necroptosis, and pyroptosis involve distinct regulated cell death processes and should not be inferred from changes in general oxidative or inflammatory markers alone [1,19].

Single-agent experimental studies provide evidence of apoptosis-related protection by volatile anesthetics, but their results cannot establish mechanisms for combinations. For example, Yao et al. [6] observed reduced terminal deoxynucleotidyl transferase dUTP nick end labeling (TUNEL) positivity following sevoflurane postconditioning in isolated rat hearts, whereas Xie et al. [5] reported reduced TUNEL positivity and caspase-3 activity following sevoflurane preconditioning in diabetic mice.

Combination studies have also directly assessed apoptosis-related endpoints. Zhang et al. [3] investigated remote ischemic preconditioning with sevoflurane postconditioning and reported a lower myocardial TUNEL apoptosis index, cleaved caspase-3, cleaved poly(ADP-ribose) polymerase (PARP), and Bax/Bcl-2 ratio in the combined-treatment group compared with either intervention alone. Arpaci et al. [12] measured renal Bax, Bcl-2, and caspase-3 immunostaining and TUNEL positivity after lower-limb I/R, observing improvements with treatment but no consistent superiority of sevoflurane plus fullerenol over fullerenol alone.

Related boric acid–propolis studies examined Bax and 8-hydroxy-2′-deoxyguanosine (8-OHdG) in renal I/R [21], and caspase-3, tumor necrosis factor-α (TNF-α), and 8-OHdG in ovarian I/R [27]. These observations concern boric acid specifically and should not be generalized to chemically distinct boron compounds or to combinations with volatile anesthetics.

Wang et al. [19] reported reductions in pyroptosis-related markers following combined ischemic and ozone postconditioning in renal I/R and cellular hypoxia–reoxygenation models. Nevertheless, marker changes and conventional group comparisons do not prove pathway necessity or synergy under a formal interaction model. Evidence for ferroptosis and necroptosis in the directly evaluated combinations remains insufficient.

### 4.5. Endothelial Dysfunction and Microcirculatory Injury

Endothelial dysfunction during reperfusion may involve glycocalyx injury, increased adhesion molecule expression, and leukocyte or platelet recruitment. These processes may impair microvascular function, although inflammatory marker expression cannot be equated with a direct measurement of microcirculatory perfusion [1].

Chappell et al. [28] examined isolated guinea pig hearts and demonstrated that sevoflurane pretreatment reduced syndecan-1 and heparan sulfate shedding, together with postischemic leukocyte and platelet adhesion. Sevoflurane was removed during reperfusion, limiting direct exposure of the subsequently infused blood cells. The study provides direct single-agent evidence of glycocalyx-associated endothelial protection rather than evidence for a therapeutic combination.

Kip et al. [10] and Arpaci et al. [12] evaluated intercellular adhesion molecule-1 (ICAM-1) immunostaining in pulmonary and renal tissue following lower-limb I/R with volatile anesthetic–fullerenol treatment. Although selected treatment groups showed lower ICAM-1 expression, combination treatment did not consistently exceed the effect of fullerenol alone. Neither study directly demonstrated changes in endothelial permeability, leukocyte adhesion in vivo, or microcirculatory blood flow. Thus, the observed ICAM-1 changes should be interpreted as endothelial-associated inflammatory findings rather than evidence that the combination preserves microcirculation.

## 5. Volatile Anesthetic-Based Combination Strategies

Direct comparisons of volatile anesthetic-based combinations are still limited. The available studies differ in species, target organ, ischemia duration, treatment timing, and which outcomes were measured. These designs allow additional benefit over each single intervention to be assessed, but not formal synergy. Most included an I/R control, the individual interventions, and a combined-treatment group, but none of the reports covered here established synergy through a prespecified interaction or additivity model.

In myocardial I/R, captopril pretreatment added to the cardioprotection seen with isoflurane preconditioning in a rabbit model, and the accompanying clinical component supported the translational relevance of the combined approach, though it did not include an isoflurane-only comparison [11]. Zhang et al. found greater protection when remote ischemic preconditioning was combined with sevoflurane postconditioning than with either alone in rats, alongside attenuation of TLR4/MyD88/NF-κB signaling [3]. These findings are consistent with additional protection, although the reported statistics do not establish synergy.

Results of the fullerenol studies were less consistent. In lower-extremity I/R models, sevoflurane or desflurane combined with fullerenol C60 reduced biochemical, inflammatory, or histological injury in several remote organs [7,8,9,10,12]. However, the combined group was not consistently superior to fullerenol alone, and the most effective arm depended on the organ and endpoint. These studies therefore support a protective effect of the individual interventions without showing a reliable incremental benefit of the combination.

Negative findings are equally informative. In isolated rabbit hearts, combining remote ischemic preconditioning with sevoflurane preconditioning provided no benefit beyond either intervention alone [13], consistent with a ceiling effect or convergence on the same downstream pathway (Section 5.3). An effective monotherapy therefore cannot be assumed to improve when paired with another protective intervention.

### 5.1. Sevoflurane and Fullerenol C60

Four studies investigated sevoflurane combined with fullerenol C60 in related lower-extremity I/R models, examining skeletal muscle [7], heart and lung [8], liver and kidney [9], and lung and kidney [12]. These studies included an I/R control, both individual interventions, and the combination. However, fullerenol dosing and sevoflurane exposure were not identical across protocols: Örnek et al. [8] reported 100 µg/kg fullerenol, whereas the other reports generally used 100 mg/kg; Arpaci et al. [12] administered sevoflurane during both ischemia and reperfusion. The combined treatment improved selected oxidative and histopathological outcomes compared with untreated I/R, but it was not consistently superior to fullerenol alone across organs and endpoints [7,8,9,12]. Arpaci et al. [12] also evaluated apoptosis-related markers, including Bax, Bcl-2, caspase-3, and TUNEL, alongside ICAM-1. None of these studies established synergy using a formal interaction model.

### 5.2. Desflurane and Fullerenol

One study tested desflurane combined with fullerenol in the same lower-extremity I/R model used for the sevoflurane–fullerenol series, looking at lung and kidney outcomes with the same four-arm design [10]. The combination reduced injury markers and tissue ICAM-1 expression relative to I/R, but, consistent with the sevoflurane–fullerenol findings, the combination was not superior to fullerenol alone, and the protective pattern differed between the two organs. Since this is the only desflurane–fullerenol study identified here, it is not yet clear whether the organ- and endpoint-dependent variability seen with sevoflurane–fullerenol is a general feature of pairing a volatile anesthetic with fullerenol, or is specific to the sevoflurane protocol.

### 5.3. Volatile Anesthetics and Ischemic Conditioning

Three studies combined a volatile anesthetic with an ischemic conditioning maneuver rather than a pharmacological adjunct, and their results were the most divergent of any subgroup. In rats, sevoflurane postconditioning combined with remote ischemic preconditioning reduced myocardial infarct size beyond either alone, with attenuation of TLR4/MyD88/NF-κB signaling proposed as a shared mechanism [3]. In isolated rat hearts, delayed remote ischemic preconditioning applied in vivo before heart isolation, combined with sevoflurane postconditioning, also produced greater protection than either intervention alone, together with higher HO-1 expression and nuclear Nrf2 translocation, again without a formal interaction test [23]. In contrast, in isolated rabbit hearts, sevoflurane preconditioning and remote ischemic preconditioning each reduced infarct size by more than 50%, but their combination provided no further benefit [13]. The positive findings came from rat models, one in vivo [3] and one using isolated hearts after in vivo limb conditioning [23], whereas the negative finding came from isolated rabbit hearts [13]. Species, conditioning protocol, timing, a ceiling effect, and limited statistical power may all contribute, and the existing studies cannot separate these explanations. This subgroup illustrates why a positive combination result in one model cannot be assumed to apply to another, even for the same organ and the same pair of interventions.

### 5.4. Isoflurane and Pharmacological Adjuncts

Tian et al. [11] evaluated captopril pretreatment combined with isoflurane preconditioning in rabbits and in patients undergoing mitral valve replacement surgery. The rabbit experiment included individual-treatment comparators and reported greater protection with combination treatment, without a formal additivity model. The clinical study included 100 patients across five captopril/isoflurane regimens, but did not include an isoflurane-only group. Favorable clinical biomarker findings therefore cannot establish added protection compared with isoflurane alone. This remains a limited example of a volatile anesthetic-based pharmacological combination reaching clinical investigation.

## 6. Summary of Direct Combination Evidence

Table 1 summarizes the 12 direct-combination studies discussed in Section 5, Section 7 and Section 8, including the experimental model, comparator structure, main finding, and whether a formal interaction assessment was performed.

## 7. Other Experimental Combination Approaches

Beyond the volatile-anesthetic combinations discussed above, two other combination strategies have been reported: ozone paired with a second conditioning maneuver, and boric acid paired with a botanical antioxidant. Neither has yet been tested together with a volatile anesthetic, although both have single-agent evidence of protection against I/R injury.

### 7.1. Combined Ozone Conditioning Strategies

Combined ischemic and ozone postconditioning reduced renal pyroptosis-related markers beyond either intervention alone in both an in vivo rat model and an in vitro hypoxia/reoxygenation model [19]. An earlier liver study compared ischemic preconditioning against ozone oxidative preconditioning, testing each on its own against an I/R control rather than combining them, and found the ozone-preconditioned group more protective on histological grounds, even though the two settings performed similarly on the biochemical measures [20]. This supports a protective effect of ozone in a second organ, but the study compared two single interventions and was not a combination study; among the studies reviewed, only the renal study [19] tested ozone together with a conditioning maneuver in the same group. Neither ozone study included a volatile anesthetic, fullerenol, or boric acid. Given the potential dose-dependent and hormetic effects of ozone conditioning [29,30], future combination studies should establish appropriate dose–response relationships rather than assume that findings from single-agent protocols can be directly transferred to combination treatments.

### 7.2. Boric Acid-Based Combinations

**Figure 2:**
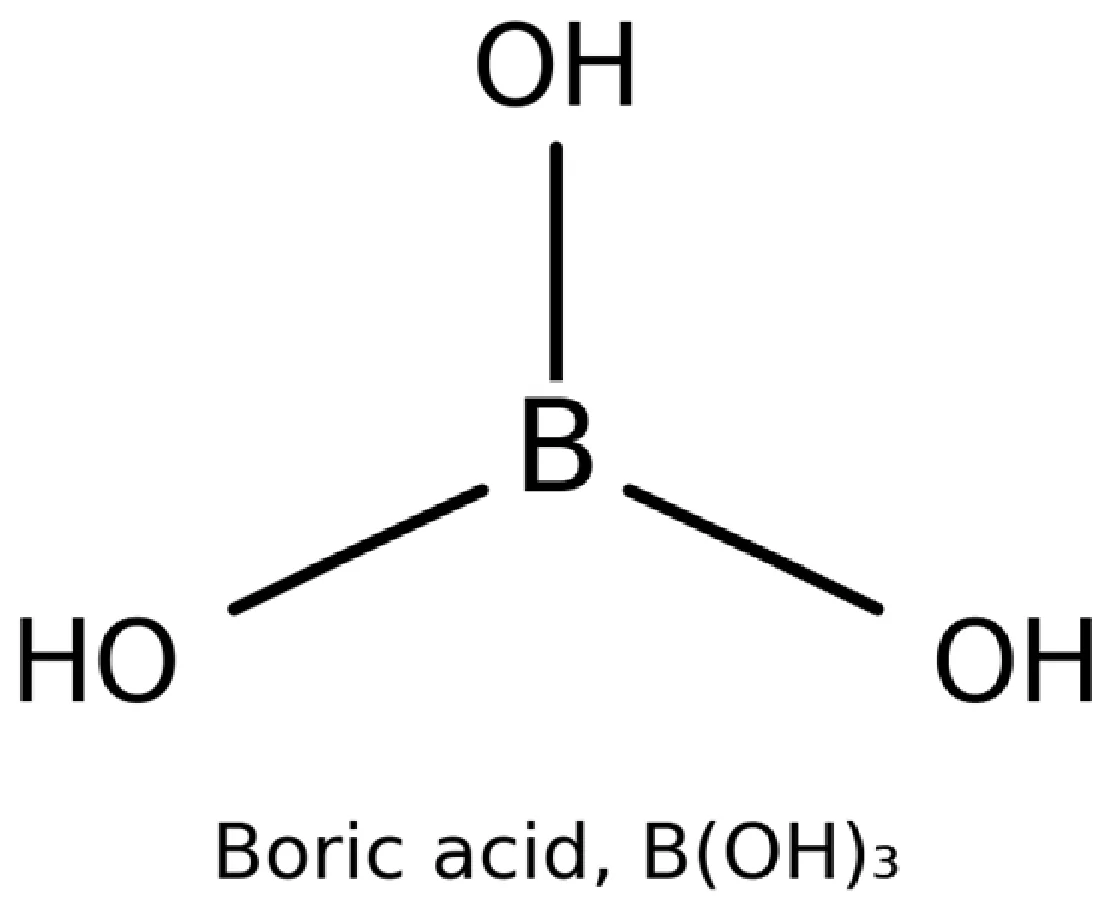
Simplified chemical structure of boric acid [B(OH)_3_]. This is the specific boron compound evaluated in the propolis combination studies discussed in Section 7.2.

Boric acid [B(OH)_3_] is a simple inorganic boron compound with a trigonal planar molecular structure. Its combination with propolis has been evaluated in separate rat renal [21] and ovarian I/R models [27]. In the renal model, propolis (200 mg/kg) and boric acid (14 mg/kg) were administered intragastrically alone or in combination before ischemia; oxidative, inflammatory, histological, Bax and 8-OHdG findings were assessed. In the ovarian model, propolis (100 mg/kg) and boric acid (7 mg/kg) were evaluated using biochemical, histological, TNF-α, caspase-3, and 8-OHdG assessments. Selected outcomes favored combined treatment, but superiority was not uniform and no formal synergy model was used. These are studies of boric acid specifically, rather than evidence for boron derivatives generally. Neither study combined boric acid with a volatile anesthetic. The simplified chemical structure of boric acid is shown in Figure 2.

## 8. Organ-Specific Evidence

Direct combination evidence varies substantially by organ and model. Volatile anesthetic-based studies have primarily investigated myocardial protection and remote organ injury following lower-limb I/R. Separate investigations evaluated ischemic conditioning combined with ozone and propolis combined with boric acid. The latter approach has also been investigated in ovarian I/R. These findings are distinguished from supporting single-treatment studies in the organ-specific sections below.

### 8.1. Heart

The heart has the most mechanistically detailed combination evidence, largely because volatile anesthetic cardioprotection is the most extensively studied single-agent mechanism in this field [5,6,23]. Isoflurane preconditioning combined with captopril produced greater cardioprotection than either intervention alone in rabbits, with supportive clinical biomarker findings in cardiac surgery patients, although the clinical part lacked an isoflurane-only comparator [11]. Sevoflurane postconditioning combined with remote ischemic preconditioning (RIPC) reduced infarct size and TLR4/MyD88/NF-κB signaling beyond either intervention alone in rats [3], and delayed RIPC combined with sevoflurane postconditioning produced similar incremental protection in isolated rat hearts, associated with higher HO-1 expression and nuclear Nrf2 translocation [23]. In contrast, the combination of sevoflurane preconditioning and RIPC provided no additional benefit in isolated rabbit hearts [13], and heart and lung outcomes in the diabetic mouse sevoflurane–fullerenol study were organ- and endpoint-dependent [8]. Myocardial evidence therefore includes both incremental protection and apparent ceiling effects when one component already provides substantial protection.

### 8.2. Brain

Cerebral I/R is the least represented organ in this combination literature. The only directly relevant study is a single-agent comparison of sevoflurane and isoflurane preconditioning in a rat model of focal cerebral ischemia, which found lower proapoptotic and lower inflammation-related transcript changes with both agents, sevoflurane showing the better profile of the two [4]. No study identified here combined a volatile anesthetic with fullerenol, ozone, or boric acid in a cerebral I/R model, and no combination study reported cerebral outcomes such as infarct volume, neurological scoring, or blood–brain barrier integrity. This is an important gap, given the relevance of ischemic stroke and of neurosurgical and cardiac procedures with a risk of cerebral hypoperfusion.

### 8.3. Liver

Hepatic evidence combines one direct fullerenol–anesthetic combination study with earlier work pairing ischemic and ozone oxidative preconditioning. The sevoflurane–fullerenol study in diabetic mice measured liver alongside kidney outcomes and found that protection varied by endpoint, with the combination not consistently better than fullerenol alone [9]. Separately, an earlier study compared ischemic preconditioning against ozone oxidative preconditioning in a hepatic I/R model, testing each on its own rather than combining them, and found the ozone-preconditioned group more protective on histological grounds; this supports ozone’s protective potential in the liver, but it is not a combination study [20]. Single-agent evidence for sevoflurane in the liver is comparatively strong, including Nrf2/HO-1 pathway activation confirmed with a pharmacological Nrf2 inhibitor [2], and a randomized trial in which sevoflurane preconditioning reduced postoperative transaminase release and complications after liver resection [31]. No boric acid combination study in the liver was found.

### 8.4. Kidney

The kidney has the broadest combination evidence of any organ, including fullerenol-, ozone-, and boric acid-based strategies. Desflurane combined with fullerenol reduced renal injury markers and ICAM-1 expression [10]; sevoflurane combined with fullerenol produced endpoint-dependent renal protection in diabetic mice [9] and in rats after lower-limb I/R [12]; propolis combined with boric acid improved oxidative, inflammatory, DNA-damage, and apoptosis-related renal outcomes [21]; and combined ischemic and ozone postconditioning reduced renal pyroptosis-related markers beyond either intervention alone [19]. Desflurane preconditioning alone has also been associated with Nrf2-Keap1 pathway changes in renal I/R [22]. The kidney therefore has a real, although methodologically limited, combination literature, and none of these renal studies used a formal interaction analysis.

### 8.5. Lung and Remote Organ Injury

Lung injury in this literature has mainly been studied as a remote-organ consequence of lower-extremity I/R rather than in direct pulmonary ischemia models. The sevoflurane–fullerenol [8,12] and desflurane–fullerenol [10] studies measured lung outcomes alongside heart or kidney endpoints; ICAM-1 reductions were reported in fullerenol-containing groups, but the combinations were not shown to be superior to fullerenol alone [10,12]. Skeletal muscle, the site of the primary ischemic insult in these models, also showed protection with sevoflurane plus fullerenol in diabetic mice [7]. Ozone preconditioning alone has been shown to protect the lung from I/R injury by attenuating NLRP3 inflammasome activation [25], but no study combined ozone with a volatile anesthetic in a pulmonary or remote-organ model. Because protection varied by organ and endpoint, the effect of a combination in one organ should not be taken as representative of its effect in others.

### 8.6. Ovary

In a rat ovarian I/R model, Geyikoglu et al. [27] investigated propolis (100 mg/kg) and boric acid (7 mg/kg), alone and in combination, administered before ischemia. Combined treatment improved selected biochemical and histopathological parameters and was associated with lower TNF-α, caspase-3, and 8-OHdG immunoreactivity. The results were not uniform across all biochemical endpoints, and no formal interaction model was applied. This study provides related boric acid–propolis evidence, not evidence for a volatile anesthetic combination.

## 9. Critical Assessment of Additive and Synergistic Claims

Across the 12 direct-combination studies summarized in Table 1, evidence for additional protection remains inconsistent. Several studies reported more favorable outcomes with combined treatment than with either intervention alone, whereas others found no clear advantage over the stronger individual intervention. These between-group comparisons do not establish additivity or synergy. Although some source articles described their results as additive or synergistic, formal reference-model interaction analyses were not reported. The available evidence should therefore be interpreted as additional, similar, or absent incremental protection, depending on the study and endpoint [15,16,17].

The results do not consistently favor combination treatment. In an isolated rabbit-heart model, remote ischemic preconditioning and sevoflurane preconditioning reduced infarct size individually, but the combination did not provide further benefit [13]. Volatile anesthetic–fullerenol studies also showed organ- and endpoint-dependent results without consistent superiority over fullerenol alone [7,8,9,10,12]. These findings may reflect overlapping protective mechanisms, treatment timing, dose selection, or limited statistical power; the available evidence does not distinguish these possibilities.

Several methodological limitations restrict mechanistic interpretation. Many studies measured oxidative stress, inflammatory markers, and histopathological outcomes without directly testing the causal contribution of the implicated pathways. More specific pathway investigations were often conducted in single-agent volatile anesthetic models rather than in the combined-treatment experiments [2,6,22,32,33]. Most combination studies were small, and formal power calculations for interaction effects were generally not reported. Future work should use appropriate control groups, clearly defined endpoints, dose–response assessments where feasible, and prespecified interaction models before describing an effect as additive or synergistic [15,16,17].

## 10. Barriers to Clinical Translation

Despite encouraging experimental findings, clinical evidence for combination strategies remains limited. Tian et al. [11] investigated captopril pretreatment with isoflurane preconditioning in patients undergoing mitral valve replacement surgery alongside a rabbit myocardial I/R model. Favorable myocardial injury and inflammatory biomarker results were reported in the clinical combination arms, but the absence of an isoflurane-only comparator precludes conclusions about added benefit beyond isoflurane alone. Thus, this constitutes limited clinical combination evidence, rather than demonstration of additivity or synergy. A separate randomized trial evaluated sevoflurane preconditioning during liver resection without a second protective intervention [31].

Indirect human evidence comes from cardiac surgery, where a Bayesian network meta-analysis of 55 randomized trials and 6921 patients found that volatile anesthesia combined with remote ischemic preconditioning was associated with a greater reduction in mortality than volatile anesthesia alone, relative to total intravenous anesthesia [34]. This finding is encouraging but does not replace a factorial trial testing volatile anesthesia, remote preconditioning, and their combination against a shared control, and it does not address fullerenol, ozone, or boric acid. A later multicenter trial that combined a volatile-anesthetic background with remote ischemic preconditioning, tight glucose control, controlled reperfusion, and other measures into one multimodal cardioprotection protocol did not reduce postoperative troponin release compared with standard care [35]. Combining several protective interventions therefore does not guarantee additional clinical benefit, even when each has independent supporting evidence. No published human trial was found that pairs a volatile anesthetic with fullerenol, ozone conditioning, or boric acid for perioperative organ protection.

### 10.1. Heterogeneity of Experimental Models

The direct-combination studies differ substantially in species (mouse, rat, rabbit), organ, ischemia duration, and outcome measures, which limits generalization of any single finding. Most experimental combination studies involved relatively small animal groups, although sample sizes and the number of animals allocated to different outcome assessments varied across studies. Many tested only one combination protocol without varying dose or timing. Diabetic and non-diabetic models are discussed in Section 10.3. No study tested these combinations in large-animal or aged-animal I/R models, and no combination study reported a power calculation for the interaction term, as opposed to the main treatment effect.

### 10.2. Dose, Timing, and Route of Administration

Dose selection and treatment timing limit comparison across studies. Most volatile anesthetic–fullerenol reports used a nominal fullerenol C60 dose of 100 mg/kg, whereas Örnek et al. [8] reported 100 µg/kg. This 1000-fold difference, which has not been reconciled in the original reports, precludes direct dose equivalence. Other important differences include the timing of administration and inconsistent route and exposure descriptions within Kip et al. [10]. In addition, incomplete chemical identification and characterization of some fullerene-based products complicate reproducibility. Future studies should define compound identity, dose, administration route, and time course explicitly. Ozone exposures require route-specific assessment, and no safety threshold for boric acid as an I/R-protective treatment can be assumed from general toxicology data [29,30,36].

### 10.3. Comorbidities and Biological Modifiers

Diabetes is the only comorbidity built into part of the volatile anesthetic–fullerenol combination literature, through the use of streptozotocin-induced diabetic rodents in the earlier sevoflurane–fullerenol studies [7,8,9], while the desflurane–fullerenol and later sevoflurane–fullerenol studies used non-diabetic rats [10,12]. This is relevant to diabetic surgical patients, but the combination effect in aged or otherwise comorbid animals remains largely unexamined, and no study directly compared the same combination across diabetic and non-diabetic animals. Sex-specific effects were not systematically reported in the combination studies covered here. In humans, the background anesthetic and any concomitant drugs can themselves change the protection being studied: the cardiac surgery network meta-analysis found that total intravenous anesthesia was linked to worse survival than volatile-agent-based anesthesia in the very comparison used to detect the anesthetic-plus-remote-preconditioning signal [34], and other clinical reports have suggested that background propofol or beta-blockade can blunt remote ischemic preconditioning specifically [37]. The clinical background against which a combination is tested, and not only the combination itself, may therefore determine whether an additional benefit can be detected.

### 10.4. Safety and Regulatory Considerations

Volatile anesthetics are the only component of these combinations with an established human safety and regulatory record, since they are already used routinely in anesthesia for purposes unrelated to organ protection; this asymmetry favors volatile anesthetic-based combinations in terms of translational feasibility. No published human pharmacokinetic, biodistribution, or clinical-safety data for fullerenol C60 were identified; the only relevant biodistribution evidence found comes from animal studies of pristine C60, which showed rapid clearance from blood with retention concentrated in the liver and spleen that persisted through the whole observation period, and hydroxylated fullerenol may differ substantially from pristine C60 in distribution and clearance [38]. Ozone therapy has a dose- and administration-dependent safety profile, and available reviews highlight the lack of standardized dosing and administration protocols. Adverse events have been reported in different clinical settings, but their frequency and relationship to specific ozone administration protocols remain insufficiently characterized [29,30]. Boric acid has a well-characterized systemic toxicology outside the I/R setting, including reproductive toxicity at high chronic exposure, but a regulatory-grade safety threshold specific to therapeutic use in I/R protection has not been established [36]. Fullerenol, ozone, and boric acid therefore each require dose-ranging and safety studies before any combination with a volatile anesthetic can move toward human testing. Recent experiments using human-derived immune cells provide additional context for evaluating fullerenol safety. Lazarev et al. [39] examined fullerenol C60(OH)24 in isolated human neutrophils and found concentration-dependent effects on viability, phagocytic activity, and reactive oxygen species production. Usanina et al. [40] studied viability, apoptosis, and metabolism in the human THP-1 monocytic cell line following fullerenol exposure. These in vitro findings underscore the importance of concentration, exposure duration, and immune-cell function in safety assessment; they do not establish clinical safety or efficacy in I/R injury, nor do they evaluate coadministration with volatile anesthetics.

## 11. Future Directions

Clinical translation requires more than favorable experimental biomarker changes. In particular, the evidence reviewed does not establish the clinical safety or efficacy of combining volatile anesthetics with fullerenol C60, ozone conditioning, or boric acid. The available clinical studies of other strategies, including perioperative cardiac conditioning and multi-component organ-protection protocols [31,34,35], cannot establish the effectiveness of the specific adjunct combinations reviewed here.

A randomized trial in liver transplantation evaluated a multi-agent pharmacological intervention comprising epoprostenol, alpha-tocopherol, melatonin, antithrombin III, infliximab, apotransferrin, recombinant erythropoietin-beta, C1-inhibitor, and glutathione [41]. The intervention did not significantly reduce peak serum aspartate aminotransferase compared with standard care. Although this trial did not investigate volatile anesthetic-based combinations, it illustrates an important translational limitation: combining agents with plausible protective mechanisms does not necessarily improve clinical outcomes. Complex multi-agent regimens also introduce additional considerations regarding administration, safety, and feasibility.

Several methodological priorities emerge from the available evidence. Future experimental studies should include appropriate I/R controls, individual-treatment groups, and combined-treatment groups, with prespecified interaction analyses and sufficient statistical power. Dose–response and timing studies are needed to define suitable treatment regimens, particularly for fullerenol and other adjunctive interventions. Studies involving aged animals, relevant comorbidities, and larger animal models may improve the assessment of biological variability and translational relevance. Further investigation of safety, pharmacokinetics, mitochondrial function, regulated cell death, endothelial permeability, and microcirculatory outcomes would also strengthen the evidence supporting future clinical studies.

## 12. Conclusions

I/R injury involves interconnected mechanisms, including oxidative stress, inflammation, mitochondrial dysfunction, regulated cell death, and endothelial injury. Combining interventions targeting different parts of this process may provide additional protection, but available experiments do not demonstrate consistent superiority over individual treatments. Studies combining volatile anesthetics with fullerenol C60, ischemic conditioning, or selected pharmacological interventions have reported favorable outcomes in some models, while others showed little or no incremental benefit. Changes in inflammatory, oxidative, apoptosis-related, and pyroptosis-associated markers do not by themselves establish a causal pathway or synergy. None of the direct-combination studies reviewed employed a formal reference-model interaction analysis.

Ozone conditioning and boric acid-based combinations provide related experimental evidence of organ protection but have not been directly investigated with volatile anesthetics in the studies discussed here. Their potential value as volatile anesthetic adjuncts therefore remains hypothetical. The distinct doses, chemical formulations, organ models, and endpoints across experiments further limit comparisons. In particular, compound identification and formulation are important for fullerene-based agents, and boric acid findings should not be generalized to chemically different boron-containing compounds.

Clinical evidence remains limited. Captopril pretreatment with isoflurane has been evaluated in patients undergoing cardiac surgery, but the absence of an isoflurane-only control constrains interpretation [11]. Before wider clinical investigation, future studies require clearly defined regimens and individual-treatment comparators, adequately powered factorial interaction analyses, dose–response and safety evaluation, and outcome measures that establish meaningful organ protection rather than isolated biochemical changes.

## Figures and Tables

**Table 1:** Direct experimental evidence for combination strategies in ischemia–reperfusion injury.

Study and Model	Combination	Comparator Structure	Main Finding	Interaction Assessment
Tian et al. [11] Rabbit myocardial I/R and cardiac surgery	Isoflurane + captopril	Complete four-arm comparison in rabbits; limited monotherapy comparison clinically	Greater protection with combined pretreatment	No formal interaction test
Zhang et al. [3] Rat myocardial I/R	Sevoflurane postconditioning + remote ischemic preconditioning	I/R, both monotherapies, and combination	Combination produced greater protection than either intervention alone	No formal interaction test
Polat et al. [7] Diabetic mouse lower-limb I/R	Sevoflurane + fullerenol C60	I/R, both monotherapies, and combination	Skeletal muscle injury reduced by combination; not uniformly superior to fullerenol alone	No formal interaction test
Şengel et al. [9] Diabetic mouse lower-limb I/R	Sevoflurane + fullerenol C60	I/R, both monotherapies, and combination	Liver and kidney protection varied by endpoint; combination was not uniformly superior	No formal interaction test
Örnek et al. [8] Diabetic mouse lower-limb I/R	Sevoflurane + fullerenol C60	I/R, both monotherapies, and combination	Heart and lung effects were organ- and endpoint-dependent	No formal interaction test
Arpaci et al. [12] Rat lower-limb I/R	Sevoflurane + fullerenol C60	I/R, both monotherapies, and combination	All treatments reduced selected injury measures; fullerenol alone was strongest for some outcomes	No formal interaction test
Kip et al. [10] Rat lower-limb I/R	Desflurane + fullerenol C60	I/R, both monotherapies, and combination	Protective effects were reported, without consistent evidence of combination superiority	No formal interaction test
Yoo et al. [13] Isolated rabbit heart	Sevoflurane preconditioning + remote ischemic preconditioning	I/R, both monotherapies, and combination	Both interventions reduced infarct size; the combination provided no additional benefit	No formal interaction test
Wang et al. [19] Rat renal I/R	Ischemic postconditioning + ozone postconditioning	I/R, both monotherapies, and combination	Combination produced greater protection and reduced pyroptosis markers	Described as synergistic, but no formal interaction model reported
Zhou et al. [23] Isolated rat heart	Sevoflurane postconditioning + delayed remote ischemic preconditioning	I/R, both monotherapies, and combination	Combination described as additive, with greater HO-1 expression and Nrf2 translocation than either alone	No formal interaction test
Geyikoglu et al. [21] Rat renal I/R	Propolis + boric acid	I/R, both monotherapies, and combination	Combination showed broad antioxidant, anti-inflammatory, and antiapoptotic protection	No formal interaction test
Geyikoglu et al. [27] Rat ovarian I/R	Propolis + boric acid	I/R, both monotherapies, and combination	Selected ovarian injury and immunohistochemical markers improved; effects varied by endpoint	No formal interaction test

Note: Treatment protocols, including doses, administration routes, and exposure durations, varied across studies. These methodological differences should be considered when interpreting and comparing the reported protective effects.

## Data Availability

No new data were created or analyzed in this study. Data sharing is not applicable to this article.
